# Supplementary material for: Resolving the full spectrum of human genome variation using Linked-Reads
Source: Genome Res. 2019 Apr;29(4):635–45. doi: 10.1101/gr.234443.118 (PMC6442396; doi:10.1101/gr.234443.118)
Supplement: Supplemental Material [file supp_gr.234443.118_Supplemental_longranger-2.2.zip › longranger-2.2.tar.bz2!/lr2.2-public/lib/stan/cmdstan/stan/doc/api/html/partials__vari_8hpp_source.html]

Stan: src/stan/agrad/partials\_vari.hpp Source File


|  |  |
| --- | --- |
|  | Stan  2.5.0  probability, sampling & optimization |


- Main Page
- Related Pages
- Namespaces
- Classes
- Files

- File List
- File Members

All Classes Namespaces Files Functions Variables Typedefs Enumerations Enumerator Friends Macros Pages

- src
- stan
- agrad

partials\_vari.hpp

Go to the documentation of this file.

1 #ifndef STAN\_\_AGRAD\_\_PARTIALS\_VARI\_HPP

2 #define STAN\_\_AGRAD\_\_PARTIALS\_VARI\_HPP

3

4 #include <stan/meta/traits.hpp>

5 #include <stan/agrad/rev/var.hpp>

6 #include <stan/agrad/rev/vari.hpp>

7

8 namespace stan {

9  namespace agrad {

10

11  class partials\_vari : public vari {

12  private:

13  const size\_t N\_;

14  vari\*\* operands\_;

15  double\* partials\_;

16  public:

17  partials\_vari(double value,

18  size\_t N,

19  vari\*\* operands, double\* partials)

20  : vari(value),

21  N\_(N),

22  operands\_(operands),

23  partials\_(partials) { }

24  void chain() {

25  for (size\_t n = 0; n < N\_; ++n)

26  operands\_[n]->adj\_ += adj\_ \* partials\_[n];

27  }

28  };

29

30  namespace {

31  template<typename T>

32  T partials\_to\_var(double logp, size\_t /\* nvaris \*/,

33  agrad::vari\*\* /\* all\_varis \*/,

34  double\* /\* all\_partials \*/) {

35  return logp;

36  }

37  template<>

38  var partials\_to\_var<var>(double logp, size\_t nvaris,

39  agrad::vari\*\* all\_varis,

40  double\* all\_partials) {

41  return var(new agrad::partials\_vari(logp, nvaris, all\_varis, all\_partials));

42  }

43

44  template<typename T,

45  bool is\_vec = is\_vector<T>::value,

46  bool is\_const = is\_constant\_struct<T>::value>

47  struct set\_varis {

48  inline size\_t set(agrad::vari\*\* /\*varis\*/, const T& /\*x\*/) {

49  return 0U;

50  }

51  };

52  template<typename T>

53  struct set\_varis <T,true,false>{

54  inline size\_t set(agrad::vari\*\* varis, const T& x) {

55  for (size\_t n = 0; n < length(x); n++)

56  varis[n] = x[n].vi\_;

57  return length(x);

58  }

59  };

60  template<>

61  struct set\_varis<agrad::var, false, false> {

62  inline size\_t set(agrad::vari\*\* varis, const agrad::var& x) {

63  varis[0] = x.vi\_;

64  return (1);

65  }

66  };

67  }

68

73  template<typename T1=double, typename T2=double, typename T3=double,

74  typename T4=double, typename T5=double, typename T6=double,

75  typename T\_return\_type=typename return\_type<T1,T2,T3,T4,T5,T6>::type>

76  struct OperandsAndPartials {

77  const static bool all\_constant = is\_constant<T\_return\_type>::value;

78  size\_t nvaris;

79  agrad::vari\*\* all\_varis;

80  double\* all\_partials;

81

82  VectorView<double\*, is\_vector<T1>::value, is\_constant\_struct<T1>::value> d\_x1;

83  VectorView<double\*, is\_vector<T2>::value, is\_constant\_struct<T2>::value> d\_x2;

84  VectorView<double\*, is\_vector<T3>::value, is\_constant\_struct<T3>::value> d\_x3;

85  VectorView<double\*, is\_vector<T4>::value, is\_constant\_struct<T4>::value> d\_x4;

86  VectorView<double\*, is\_vector<T5>::value, is\_constant\_struct<T5>::value> d\_x5;

87  VectorView<double\*, is\_vector<T6>::value, is\_constant\_struct<T6>::value> d\_x6;

88

89  OperandsAndPartials(const T1& x1=0, const T2& x2=0, const T3& x3=0,

90  const T4& x4=0, const T5& x5=0, const T6& x6=0)

91  : nvaris(!is\_constant\_struct<T1>::value \* length(x1) +

92  !is\_constant\_struct<T2>::value \* length(x2) +

93  !is\_constant\_struct<T3>::value \* length(x3) +

94  !is\_constant\_struct<T4>::value \* length(x4) +

95  !is\_constant\_struct<T5>::value \* length(x5) +

96  !is\_constant\_struct<T6>::value \* length(x6)),

97  all\_varis((agrad::vari\*\*)agrad::chainable::operator new(sizeof(agrad::vari\*) \* nvaris)),

98  all\_partials((double\*)agrad::chainable::operator new(sizeof(double) \* nvaris)),

99  d\_x1(all\_partials),

100  d\_x2(all\_partials

101  + (!is\_constant\_struct<T1>::value) \* length(x1)),

102  d\_x3(all\_partials

103  + (!is\_constant\_struct<T1>::value) \* length(x1)

104  + (!is\_constant\_struct<T2>::value) \* length(x2)),

105  d\_x4(all\_partials

106  + (!is\_constant\_struct<T1>::value) \* length(x1)

107  + (!is\_constant\_struct<T2>::value) \* length(x2)

108  + (!is\_constant\_struct<T3>::value) \* length(x3)),

109  d\_x5(all\_partials

110  + (!is\_constant\_struct<T1>::value) \* length(x1)

111  + (!is\_constant\_struct<T2>::value) \* length(x2)

112  + (!is\_constant\_struct<T3>::value) \* length(x3)

113  + (!is\_constant\_struct<T4>::value) \* length(x4)),

114  d\_x6(all\_partials

115  + (!is\_constant\_struct<T1>::value) \* length(x1)

116  + (!is\_constant\_struct<T2>::value) \* length(x2)

117  + (!is\_constant\_struct<T3>::value) \* length(x3)

118  + (!is\_constant\_struct<T4>::value) \* length(x4)

119  + (!is\_constant\_struct<T5>::value) \* length(x5))

120  {

121  size\_t base = 0;

122  if (!is\_constant\_struct<T1>::value)

123  base += set\_varis<T1>().set(&all\_varis[base], x1);

124  if (!is\_constant\_struct<T2>::value)

125  base += set\_varis<T2>().set(&all\_varis[base], x2);

126  if (!is\_constant\_struct<T3>::value)

127  base += set\_varis<T3>().set(&all\_varis[base], x3);

128  if (!is\_constant\_struct<T4>::value)

129  base += set\_varis<T4>().set(&all\_varis[base], x4);

130  if (!is\_constant\_struct<T5>::value)

131  base += set\_varis<T5>().set(&all\_varis[base], x5);

132  if (!is\_constant\_struct<T6>::value)

133  set\_varis<T6>().set(&all\_varis[base], x6);

134  std::fill(all\_partials, all\_partials+nvaris, 0);

135  }

136

137  T\_return\_type

138  to\_var(double logp) {

139  return partials\_to\_var<T\_return\_type>(logp, nvaris, all\_varis, all\_partials);

140  }

141  };

142

143  }

144 }

145

146

147 #endif

stan::agrad::OperandsAndPartials::all\_varis

agrad::vari \*\* all\_varis

**Definition:** partials\_vari.hpp:79

stan::is\_constant

Metaprogramming struct to detect whether a given type is constant in the mathematical sense (not the ...

**Definition:** traits.hpp:43

vari.hpp

stan::agrad::OperandsAndPartials::to\_var

T\_return\_type to\_var(double logp)

**Definition:** partials\_vari.hpp:138

stan::agrad::chainable

Abstract base class for variable implementations that handles memory management and applying the chai...

**Definition:** chainable.hpp:14

stan::length

size\_t length(const T &)

**Definition:** traits.hpp:159

stan::agrad::OperandsAndPartials::OperandsAndPartials

OperandsAndPartials(const T1 &x1=0, const T2 &x2=0, const T3 &x3=0, const T4 &x4=0, const T5 &x5=0, const T6 &x6=0)

**Definition:** partials\_vari.hpp:89

stan::agrad::OperandsAndPartials

A variable implementation that stores operands and derivatives with respect to the variable...

**Definition:** partials\_vari.hpp:76

traits.hpp

stan::is\_constant\_struct

Metaprogram to determine if a type has a base scalar type that can be assigned to type double...

**Definition:** traits.hpp:57

stan::agrad::OperandsAndPartials::d\_x2

VectorView< double \*, is\_vector< T2 >::value, is\_constant\_struct< T2 >::value > d\_x2

**Definition:** partials\_vari.hpp:83

stan::agrad::partials\_vari::chain

void chain()

Apply the chain rule to this variable based on the variables on which it depends. ...

**Definition:** partials\_vari.hpp:24

stan::math::fill

void fill(T &x, const S &y)

Fill the specified container with the specified value.

**Definition:** fill.hpp:22

stan::agrad::vari

The variable implementation base class.

**Definition:** vari.hpp:28

stan::agrad::OperandsAndPartials::d\_x6

VectorView< double \*, is\_vector< T6 >::value, is\_constant\_struct< T6 >::value > d\_x6

**Definition:** partials\_vari.hpp:87

var.hpp

stan::agrad::OperandsAndPartials::all\_partials

double \* all\_partials

**Definition:** partials\_vari.hpp:80

stan::agrad::OperandsAndPartials::nvaris

size\_t nvaris

**Definition:** partials\_vari.hpp:78

stan::agrad::OperandsAndPartials::all\_constant

static const bool all\_constant

**Definition:** partials\_vari.hpp:77

stan::agrad::OperandsAndPartials::d\_x4

VectorView< double \*, is\_vector< T4 >::value, is\_constant\_struct< T4 >::value > d\_x4

**Definition:** partials\_vari.hpp:85

stan::agrad::OperandsAndPartials::d\_x5

VectorView< double \*, is\_vector< T5 >::value, is\_constant\_struct< T5 >::value > d\_x5

**Definition:** partials\_vari.hpp:86

stan::agrad::OperandsAndPartials::d\_x1

VectorView< double \*, is\_vector< T1 >::value, is\_constant\_struct< T1 >::value > d\_x1

**Definition:** partials\_vari.hpp:82

stan::agrad::OperandsAndPartials::d\_x3

VectorView< double \*, is\_vector< T3 >::value, is\_constant\_struct< T3 >::value > d\_x3

**Definition:** partials\_vari.hpp:84

stan::agrad::partials\_vari::partials\_vari

partials\_vari(double value, size\_t N, vari \*\*operands, double \*partials)

**Definition:** partials\_vari.hpp:17

stan::VectorView

VectorView is a template metaprogram that takes its argument and allows it to be used like a vector...

**Definition:** traits.hpp:275

stan::agrad::vari::adj\_

double adj\_

The adjoint of this variable, which is the partial derivative of this variable with respect to the ro...

**Definition:** vari.hpp:43

stan::agrad::partials\_vari

**Definition:** partials\_vari.hpp:11


---

[ Stan Home Page ]

*© 2011–2014,
Stan Development Team.*
